# Supplementary material for: Intracellular Fate of Sub-Toxic Concentration of Functionalized Selenium Nanoparticles in Aggressive Prostate Cancer Cells
Source: Nanomaterials (Basel). 2023 Nov 22;13(23):2999. doi: 10.3390/nano13232999 (PMC10707848; doi:10.3390/nano13232999)
Supplement: Supplementary file 1 [file nanomaterials-13-02999-s001.zip › nanomaterials-2694931-supplementary.pdf]

Supplementary materials for

Intracellular Fate of Sub-Toxic Concentration of Functionalized Selenium Nanoparticles in Aggressive Prostate Cancer Cells

Caroline Bissardon[1,*], Olivier Proux[2], Salvatore Andrea Gazze[3], Odile Filhol[4], Benoît Toubhans[3], Lucie Sauzéat[5], Sylvain Bouchet[5], Aled R. Lewis[3], Thierry Maffeis[3], Jean-Louis Hazemann[6], Sam Bayat[1], Peter Cloetens[7], R. Steve Conlan[3], Laurent Charlet[8] and Sylvain Bohic[1,7,*]

[1] Université Grenoble Alpes, Inserm, UA7, Synchrotron Radiation for Biomedicine (STROBE), 38043 Grenoble, France

[2] OSUG, UAR 832 CNRS, Université Grenoble Alpes, IRD, INRAe, Météo- France, 38041 Grenoble, France

[3] Swansea University Medical School, Swansea University, Swansea SA2 8PP, UK

[4] Laboratoire de Biologie et Biotechnologies pour la Santé, IRIG-DS, Inserm U1292, CEA, Université Grenoble-Alpes, 38054 Grenoble, France

[5] Institute of Biogeochemistry and Pollutant Dynamics, ETH Zurich, CH-8092 Zurich, Switzerland.

[6] Institut Néel CNRS-UGA, 25 Avenue des Martyrs, 38042 Grenoble, France

[7] ESRF—The European Synchrotron Radiation Facility, 38043 Grenoble, France

[8] ISTerre, Université Grenoble Alpes, 38041 Grenoble, France;

* Correspondence: cbissardon@embl.fr, sylvain.bohic@inserm.fr

1. XANES calculations

Table S1: Crystallographic structures used for the XANES calculations of Se^0 components. Calculations were performed taking into account cluster radii $\Re$ equals to 3, 4, 5 and 6 Å.

Ref.	Form.	Space Group	Name	Crystallographic scheme
[1]	Se Grey	P3121	Se-6<br>Trigonal	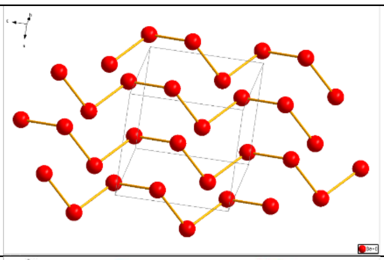
[2]	Se-6	R3-H	Se-6<br>Rhombohedral	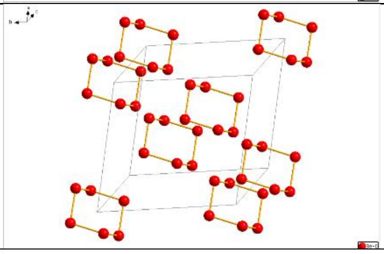
[3]	Se Red	P121/N1	Se-8<br>Monoclinic	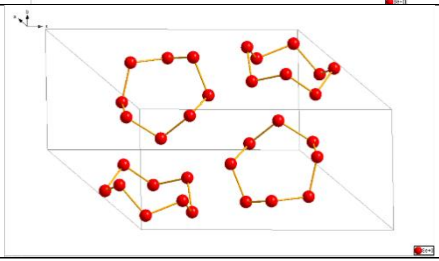

Figure S1: Experimental and calculated spectra for the determination of the Se speciation in the BSA and chitosan nanoparticles, as received and after incubation, by XANES least-square fitting using Se^{IV} experimental and Se^{0} calculated spectra as references: systematic study of the influence of both the radius of calculation ($\Re$) and the crystallographic structure (monoclinic, rhombohedric or trigonal) used for the Se^{0} calculation. R-factor of the best fits are in bold for better clarity.

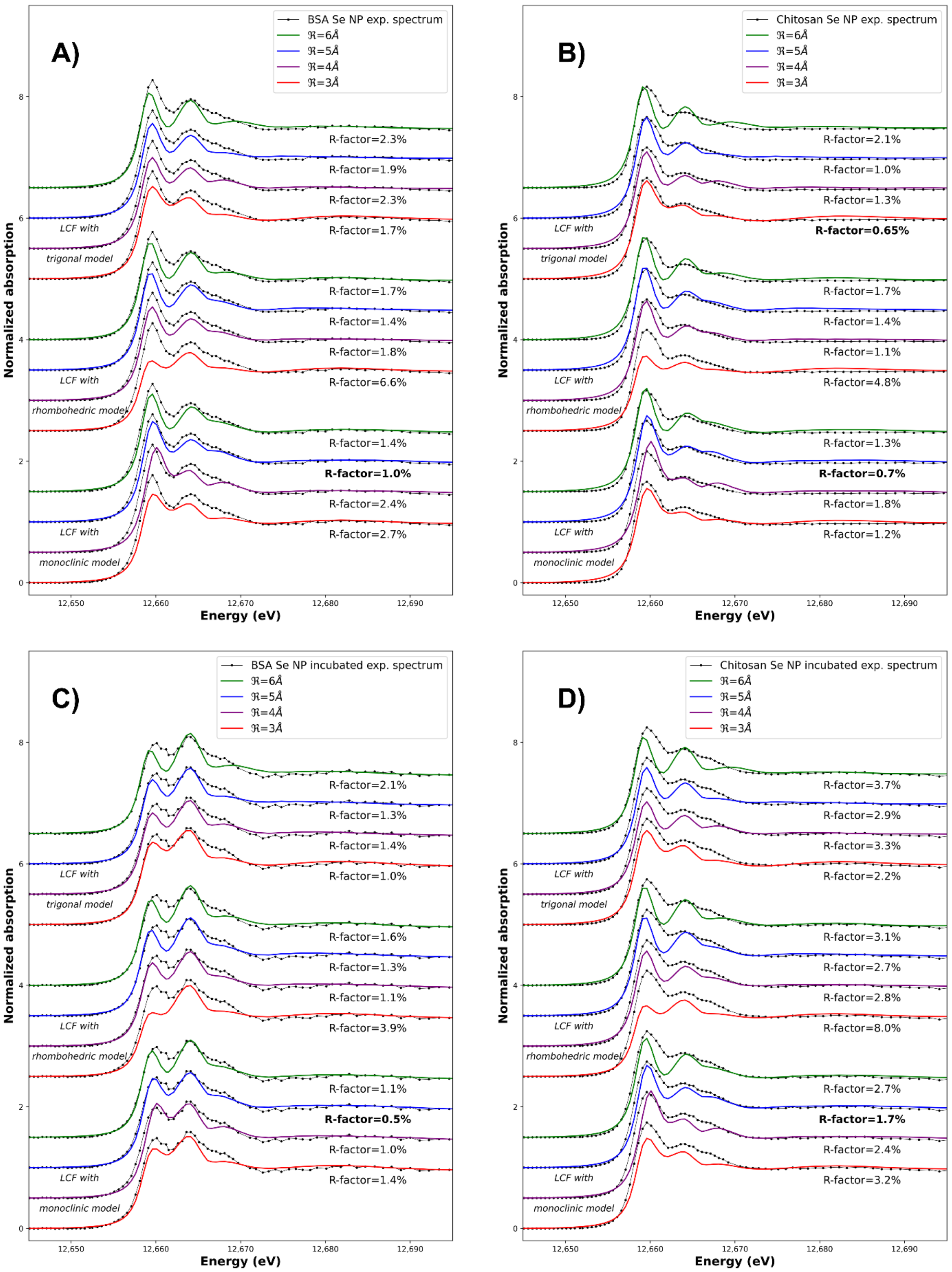

Table S2: Values obtained on the determination of the Se speciation in the BSA and chitosan nanoparticles, as received and after incubation, by XANES least-square fitting using Se^{IV} experimental and Se^{0} calculated spectra as references: systematic study of the influence of both the radius of calculation ($\Re$) and the crystallographic structure (monoclinic, rhombohedric or trigonal) used for the Se^{0} calculation. Best fits values are in bold for better clarity.

**BSA Se NP**				**Chitosan Se NP**			
	$\Re$ (Å)	Se^{0} calc.	Se^{IV} exp.		$\Re$ (Å)	Se^{0} calc.	Se^{IV} exp.
Trigonal model	6	65.4%	34.6%	*Trigonal model*	6	71.1%	28.9%
	5	69.1%	30.9%		5	75.6%	24.4%
	4	69.8%	30.2%		4	76.4%	23.6%
	3	77.0%	23.0%		**3**	**84.1%**	**15.9%**
Rhombohedric model	6	66.8%	33.2%	*Rhombohedric model*	6	72.4%	27.6%
	5	68.5%	31.5%		5	74.3%	25.7%
	4	71.6%	28.4%		4	78.1%	21.9%
	3	79.3%	20.7%		3	88.7%	11.3%
Monoclinic model	6	72.1%	27.9%	*Monoclinic model*	6	78.2%	21.8%
	**5**	**71.3%**	**28.7%**		**5**	**77.4%**	**22.6%**
	4	69.6%	30.4%		4	75.9%	24.1%
	3	77.6%	22.4%		3	85.3%	14.7%
**BSA Se NP incubated**				**Chitosan Se NP incubated**			
	$\Re$ (Å)	Se^{0} calc.	Se^{IV} exp.		$\Re$ (Å)	Se^{0} calc.	Se^{IV} exp.
Trigonal model	6	54.3%	45.7%	*Trigonal model*	6	66.6%	33.4%
	5	57.9%	42.1%		5	70.8%	29.2%
	4	58.6%	41.4%		4	71.4%	28.6%
	3	64.5%	35.5%		3	79.1%	20.9%
Rhombohedric model	6	55.5%	44.5%	*Rhombohedric model*	6	68.1%	31.9%
	5	57.1%	42.9%		5	69.9%	30.1%
	4	60.0%	40.0%		4	73.2%	26.8%
	3	67.2%	32.8%		3	81.0%	19.0%
Monoclinic model	6	60.2%	39.8%	*Monoclinic model*	6	73.6%	26.4%
	**5**	**59.8%**	**40.2%**		**5**	**73.3%**	**26.7%**
	4	58.8%	41.2%		4	72.0%	28.0%
	3	65.3%	34.7%		3	79.7%	20.3%

Optimum adjustments are always obtained for $\Re$ =5Å and a monoclinic structure, as for the Se^{0} red reference, except for Chitosan Se NP where an adjustment of slightly better quality is obtained for $\Re$ =3Å and a trigonal structure. Two points factors lead us not to consider this solution as it would induce:

- a structural evolution (from trigonal to monoclinic) with incubation,
- an improvement of the crystallinity of the Se^{0} core as the corresponding $\Re$ radius would increase from 3 to 5Å.

2. Cellular Se concentration

Table S3: Cellular Se concentrations and speciation measured on a QQQ-ICP-MS (Agilent 8900). n/a stands for non-measured sample. <LD stands for below the limit of detection calculated following IUPAC guidelines as $LD_i = x_{bi} + k\ s_{bi}$ where k = 3 (95% confidence level), and x_{bi} and s_{bi} are, respectively, the mean and standard deviation of the number of counts measured in blanks (i.e. acids stored and handled with the same material used to test the samples). n include replicate data only (i.e. re-run analyses)

	Culture media = supernatant								Cell pellet = solid residue		
	Total Se measured on digested samples			Se speciation [ppb, n=1] measured on non-digested samples					Total Se measured on digested samples		
	**Se (ppb) [wet mass]**	**2sd**	**n**	**SeCys**	**SeIV**	**SeMet**	**Unk.**	**SeVI**	**Se [ng/cells]**	**2sd**	**n**
Ctrl-PC3-1	7.41	0.25	2	0.014	0.036	0.001	1.058	0.001	3.44E-06	n/a	1
Ctrl-PC3-2	n/a	n/a	n/a	0.004	<LD	<LD	1.008	0.003	1.13E-06	n/a	1
Ctrl-PC3-3	8.61	0.71	2	0.036	0.584	0.005	1.018	0.005	n/a	n/a	n/a
Ctrl-PC3-4	n/a	n/a	n/a	0.010	0.011	0.006	1.029	<LD	n/a	n/a	n/a
SeIV-PC3-1	483.54	8.51	2	10.116	86.510	0.644	1.204	0.188	8.97E-05	n/a	1
SeIV-PC3-2	448.48	6.42	2	9.373	72.183	0.552	1.232	0.096	1.00E-04	n/a	1
SeIV-PC3-3	454.86	7.35	2	9.523	71.232	0.685	1.050	0.013	3.64E-05	3.18E-06	2
SeIV-PC3-4	336.01	4.09	2	6.371	78.879	0.034	0.852	0.069	4.63E-05	9.66E-07	2
BSA-PC3-1	540.39	6.55	2	9.695	93.772	0.107	1.122	2.161	4.88E-05	1.39E-06	2
BSA-PC3-2	525.88	9.88	2	9.382	113.452	0.715	1.022	2.103	4.72E-05	1.11E-06	2
BSA-PC3-3	420.09	12.11	2	7.345	109.090	0.455	1.106	1.739	7.89E-05	2.63E-06	2
BSA-PC3-4	518.56	5.87	2	9.775	61.578	<LD	1.345	2.272	4.01E-05	2.32E-06	2
Chito-PC3-1	80.93	0.47	2	1.016	16.542	0.083	1.028	1.268	1.62E-06	3.01E-07	2
Chito-PC3-2	59.25	0.52	2	0.669	16.559	0.025	0.652	0.991	2.30E-06	4.64E-07	2
Chito-PC3-3	53.83	2.03	2	0.579	11.661	0.006	0.536	0.824	8.64E-07	1.07E-07	2
Chito-PC3-4	82.25	0.03	2	1.049	15.601	0.063	1.036	1.274	9.36E-07	1.49E-07	2

Table S4: Selenium concentrations of Se-enriched yeast certified reference material (SELM-1)

Reference material - SELM1			
**Sample Name**	**Se (ppm) [dry mass]**	**2sd**	**n**
SELM-1	1994	61	3
SELM-2	1981	59	4
SELM-3	1941	55	4
SELM-4	1877	162	3
**Certified value (NRC) SELM-1**	**2031**	**70**	

3. Wound healing assay

Figure S2: High resolution figure wound healing assay of PC3-cells at time 0, 10 and 24h (corresponds to Figure 7.D of the article). Selenium compounds (0µg/ml) was used as the control. Magnification: x10. Red rectangles schematically show the area of the wound (T0), and the regions of proliferation/migration (T10/T24).

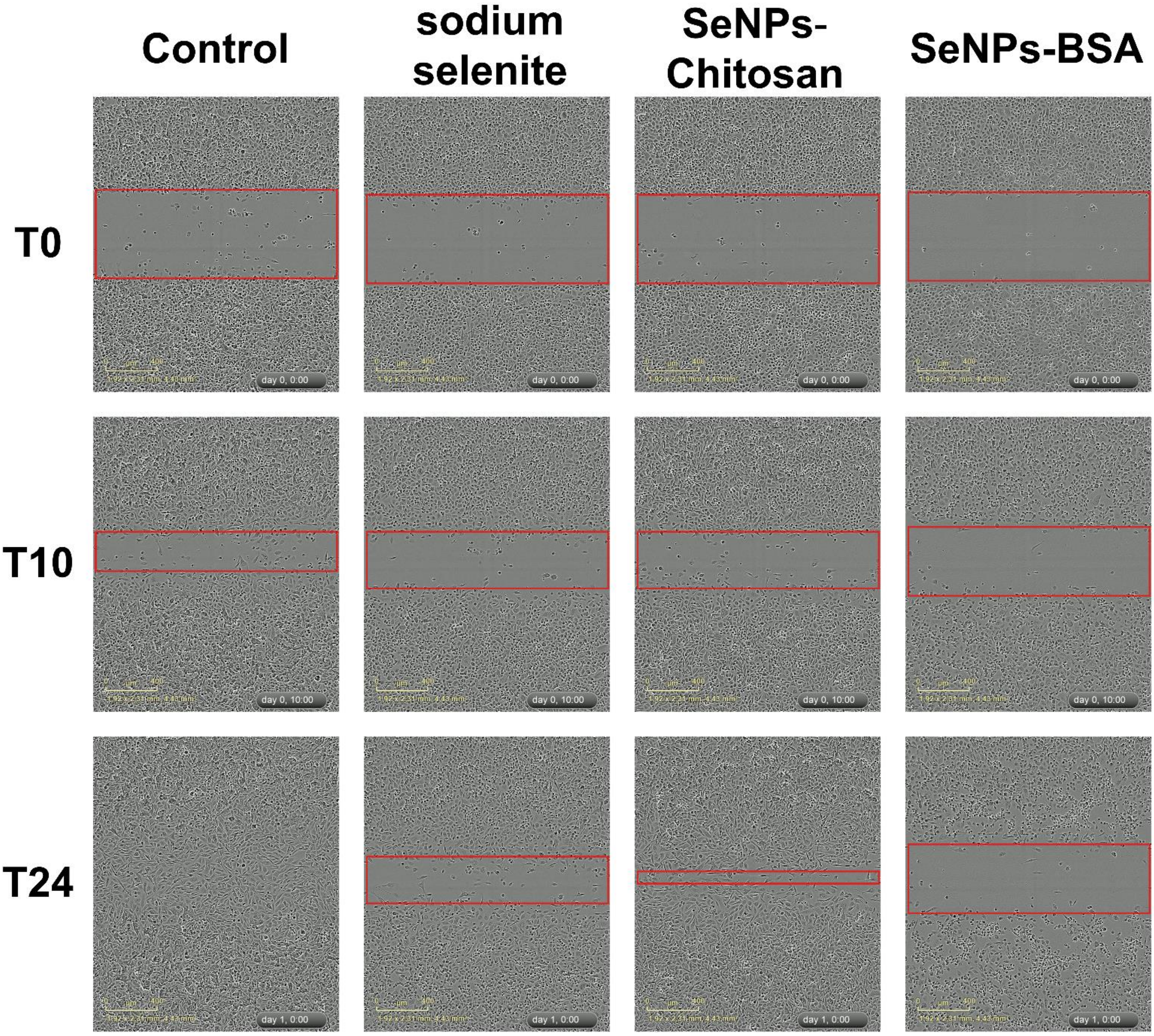

4. X-ray fluorescence elemental mapping

Figure S3: Representative X-ray fluorescence imaging at 200nm stepzize at I14 Diamond nanoprobe beamline for plunge-frozen/Freeze-dried PC-3 cells exposed 24h at an IC20 concentration of SeNPs-BSA, SeNPs-Chitosan and sodium selenite aqueous solution. Control PC-3 cells are without any Se treatment. The cellular distribution for potassium (K), phosphorus (P), sulfur (S), selenium (Se) and zinc (Zn) are presented. n.d.: not detected.

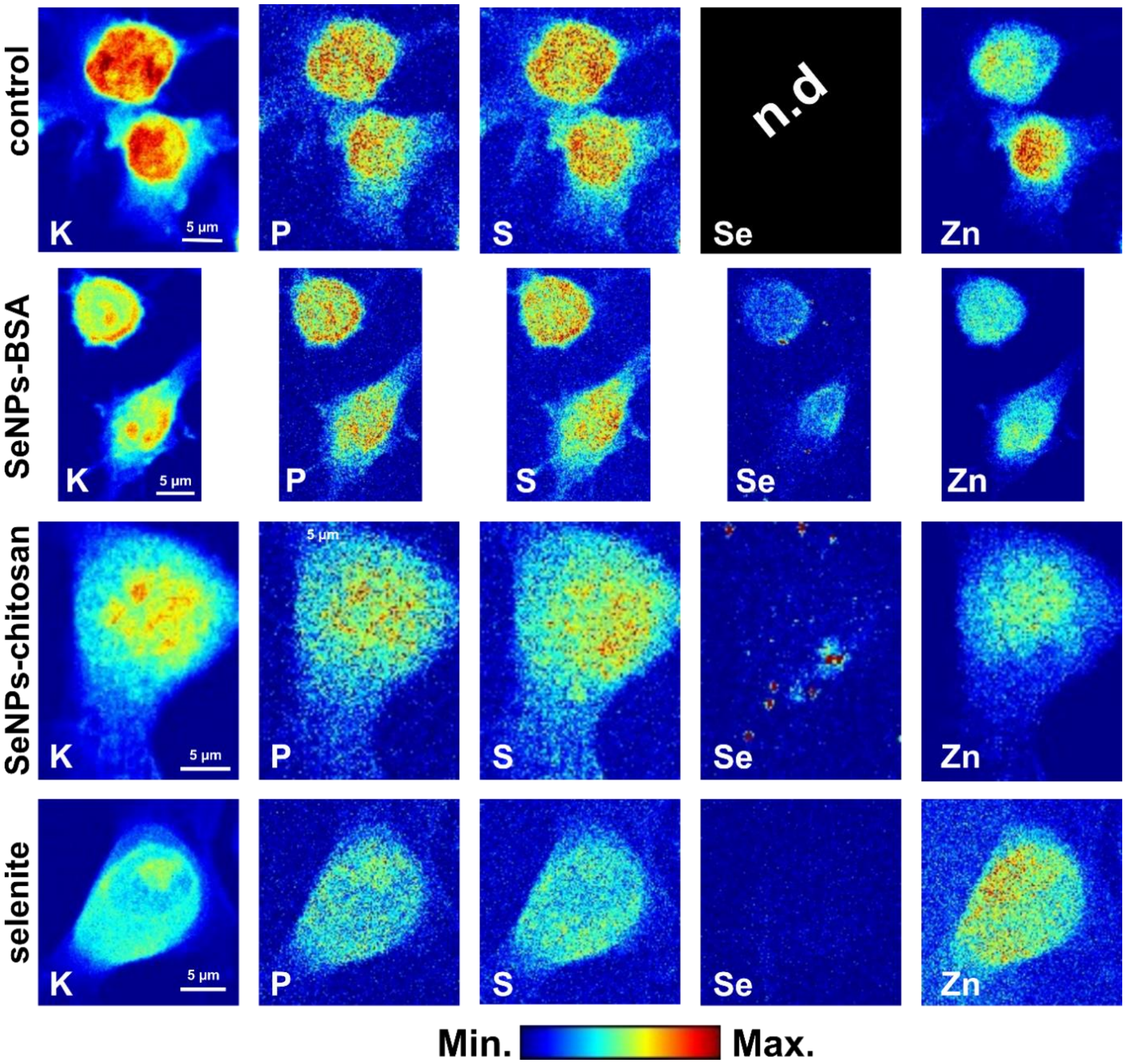

Figure S4. Cryo-correlative imaging of PC-3 cells exposed to IC20 concentration of SeNPs-Chitosan for 24h. A) Cryo-optical fluorescence imaging (120K) of vitrified PC-3 cells cultured on Si3N4 membranes. Live cells were first incubated overnight with Lysosomes-RFP CellLight™, BacMam 2.0 reagent following manufacturer recommendations, then cells were exposed for 24h to SeNPs-Chitosan at IC20 concentration, followed by plunge-freezing procedure. B) Zoom in the insert of A). C) Hard X-ray phase contrast imaging of the same cells (panel B) at the ID16A cryo-nanoimaging allow to show some structural details mainly the nuclear region and shape of the cell. D) Merge of different element maps (Potassium K - blue, Selenium Se - red, Zinc Zn - green) of the same cells (panel B). K attests of the preservation of cell integrity after freezing. E) Cryo-XRF nanoimaging at 50nm step-size of a panel of different element maps (Phosphorus P, Selenium Se, Zinc Zn and Sulfur S) distribution in the same PC-3 cells exposed to SeNPs-BSA as panel B.

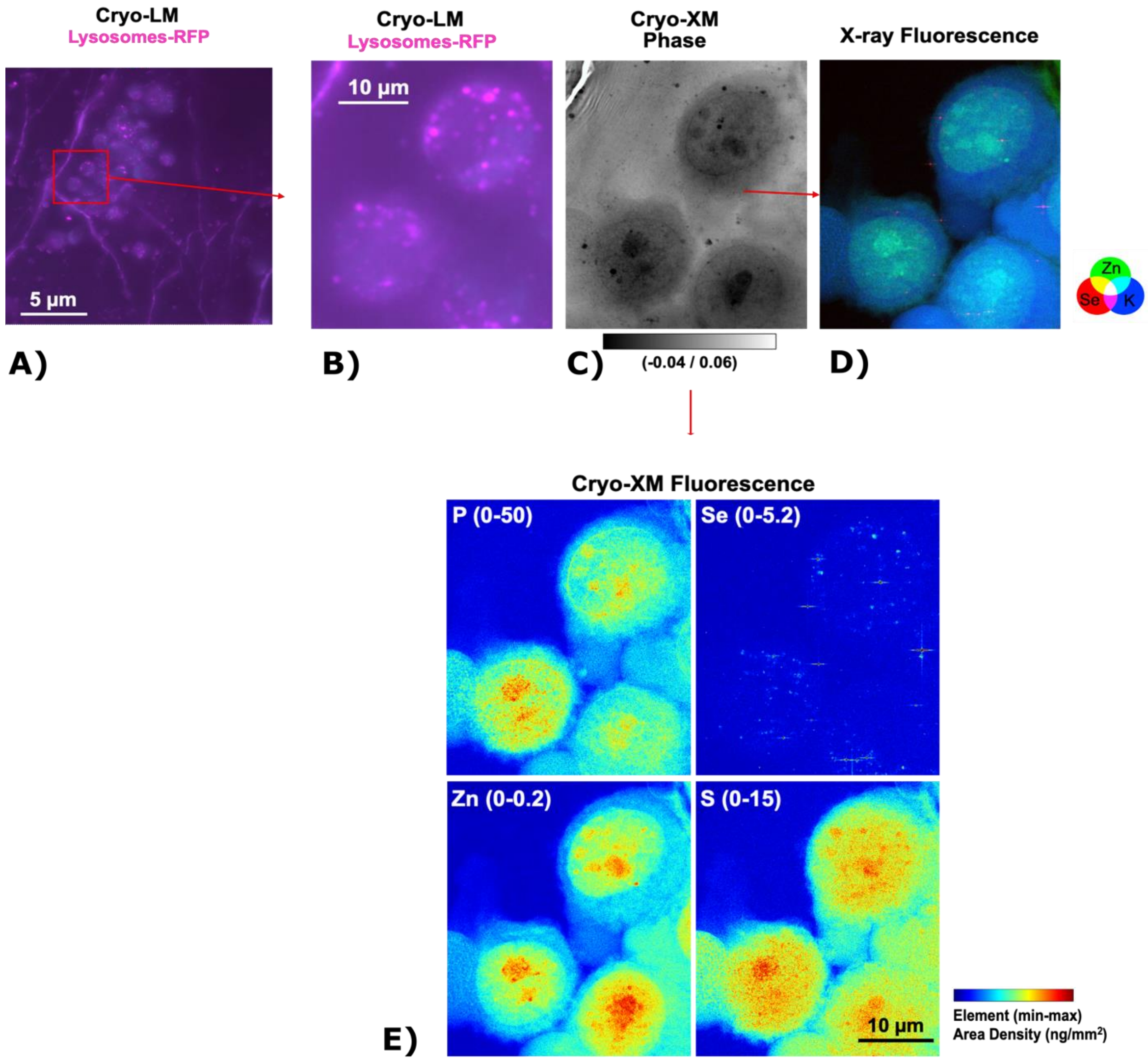

4. Genotoxicity

4.1. Genotoxicity Assessment of selenium nanoparticles on cancer cells

At day 1, 20 µL of cell solution were plated onto 12-mm diameter coverslip (20000 cells/20µL). After 5min at 37°C, 2 mL medium are added to the cells and left overnight in the incubator (37°C, 5% CO2). At day 2, the medium and cell debris were removed and replaced by fresh new medium containing SeNPs-BSA or Chitosan or sodium selenite. A concentration range [0.1, 1, 10, 100 µM(Se)] was used. At day 3, after 24h incubation, cells were thoroughly rinsed 3 times with PBS. Further, the 2 Gy γ-irradiation was performed. Dosimetry certifications and the γ-rays irradiations were performed with a 6 MeV γ-rays clinical irradiator (SL 15 Phillips) (dose-rate: 6 Gy.min-1) at anti-cancer Centre Léon-Bérard, (Lyon, France) [4, 5]. Control followed the same stages than treated cells except the irradiation. For the kinetic analysis, cells were fixed with 100µL solution of 4% PFA diluted in PBS for 10 min and rinsed with twice with PBS. Next, Immunofluorescence protocol and foci scoring were followed as described elsewhere [4, 6]. The stabilization was obtained with 200µL of lysis solution laid on cells for 3 min, and then rinsed with PBS. Coverslips were transferred to a new petri dish. Anti-γ-H2AXser139 antibody (clone JBW301; Merck, Millipore, Darmstadt, Germany) was applied at 1:800. Anti-pATMser1981 (clone 10H11.E12; Millipore, Germany) antibodies were used at 1:100. The first antibody, pH2AX, was used in a 1:800 dilution. 30µL were used per coverslip, incubation at 37°C for 1h, then rinsed with PBS. In a light-preserved room, 30 µL of second Ab, diluted in 1:100 in PBS-3%BSA solution, was applied on coverslips containing cells (20min at 37°C), then rinsed with PBS. Cells on the coverslips are labelled with Vectashield DAPI (H100 abcys) mounting solution. DAPI allows to see micronuclei and DNA.

4.2. Genotoxocity assay

We studied the induced DNA damage (double-strand breaks - DSBs) by γ-H2AX labeling and micronucleus labeling. The kinetics of nuclear pATM foci (repair signaling protein) are used to quantify DSB recognition prior to repair by suturing (the predominant repair mode in mammals). Se^0NPs or sodium selenite do not generate a significant number of DSBs and, consequently, the rate of persistent DSBs after 24h of exposure remains very low at the concentrations we had chosen, but also for high concentrations such as of 100 µM. At sub-cytotoxic concentrations, the notion of genotoxicity of the soluble sodium selenite species are non-existent and there are no major effects concerning the 2 types of nanoparticles. Figure S4 shows a slowdown in the rate of recognition by DSBs generated by pre-irradiation of PC3 cells following 24h exposure to BSA-coated Se°NPs, which (above) caused a slowdown in the proliferation of these highly aggressive prostate cancer cells. This effect on ATM suggests indirectly that internalized and potentially biotransformed Se°NPs have an impact intracellularly, leading to a reduction in the migration and hence invasion capacity of these aggressive cells, but also to a slowing down of ATM addressing to the nucleus when DSBs are generated.

Figure S5: Plot of DSB number (ph) and micronucleus number (µN) for PC-3 cells exposed to increasing concentrations of 0 (control) to 100 µM Se°NPs coated with BSA (left) or chitosan (middle). The curves on the right show DSB repair time after irradiation at 2Gy (generating 70-75 double-strand breaks) following exposure to SeNPs or sodium selenite salts (10µM for 24h).

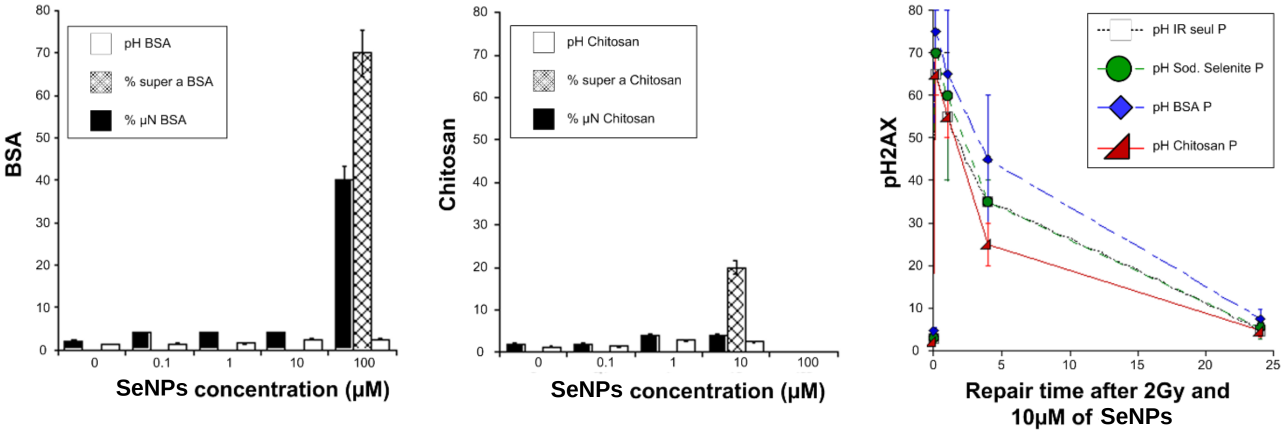

5. References

1. Cherin, P.; Unger, P. The crystal structure of trigonal selenium, *Inorg. Chem.* **1967**, *6*, 1589-1591. https://dx.doi.org/10.1021/ic50054a037

2. Miyamoto, Y. Structure and phase transformation of rhombohedral selenium composed of Se_6 molecules. *Jpn. J. Appl. Phys.* **1980**, *19*, 1813-1819. https://doi.org/10.1143/JJAP.19.1813

3. Maaninen, A.; Konu, J.; Laitinen, R.S.; Chivers, T.; Schatte, G.; Pietikainen, J.; Ahlgren, M. Preparation, crystal structure, and spectroscopic characterization of $((Se_2SN_2)Cl)_2$. *Inorg. Chem.* **2001**, *40*, 3539-3543. https://dx.doi.org/10.1021/ic010141h

4. Granzotto, A. *et al.* Influence of Nucleoshuttling of the ATM Protein in the Healthy Tissues Response to Radiation Therapy: Toward a Molecular Classification of Human Radiosensitivity. *Int. J. Radiat. Oncol. Biol. Phys.* **2016**, 94, 450--460. doi: 10.1016/J.IJROBP.2015.11.013.

5. Ferlazzo, M.L.; Sonzogni, L.; Granzotto, A.; Bodgi, L.; Lartin, O.; Devic, C.; Vogin, G.; Pereira, S.; Foray N. Mutations of the Huntington's disease protein impact on the ATM-dependent signaling and repair pathways of the radiation-induced DNA double-strand breaks: Corrective effect of statins and bisphosphonates. *Mol. Neurobiol*. **2014**, *49*, 1200–1211. https://doi.org/10.1007/s12035-013-8591-7.

6. Foray, N.; Marot, D.; Gabriel, A.; Randrianarison, V.; Carr, A.M.; Perricaudet, M.; Ashworth, A.; Jeggo, P. A subset of atm- and atr-dependent phosphorylation events requires the brca1 protein. *EMBO J.* **2003**, *22*, 2860-2871. https://doi.org/10.1093/emboj/cdg274.
